# Telepresence avatar use for children with cancer in schools: a study on barriers and facilitators

**DOI:** 10.3389/fdgth.2026.1867390

**Published:** 2026-07-09

**Authors:** Friederike Carlotta Grabowski, Jana Dördelmann, Britta Exner, Fabian Simon Frielitz

**Affiliations:** Department of Telemedicine, Digitalization and Economics in Medicine, Medical Faculty, Otto-von-Guericke University Magdeburg, Magdeburg, Germany

**Keywords:** cancer, children and adolescents, digital health, implementation barriers, pediatric oncology, telepresence avatar

## Abstract

**Purpose:**

Children with cancer often face psychosocial and educational challenges due to prolonged school absences. Telepresence avatars offer the possibility of maintaining virtual classroom participation. This study examines perceived barriers and facilitators to the implementation of telepresence avatars in schools from the perspectives of teachers and supplements these findings with parent perspectives.

**Methods:**

A cross-sectional teacher survey was conducted, complemented by an exploratory qualitative parent-interview component. For the survey, 5,019 schools were contacted via e-mail. Teachers' perceptions were assessed via a quantitative questionnaire, including descriptive analyses and group comparisons based on experience with pediatric cancer and/or prior use of telepresence avatars. To illustrate implementation experiences from the family perspective, three parents of children with cancer who had used a telepresence avatar were interviewed.

**Results:**

Teachers (*N* = 298) reported moderate technical and organizational barriers to telepresence avatar use. Significant differences in perceived barriers were observed across experience profiles, with the lowest overall barrier levels reported by teachers with combined experience in pediatric cancer and telepresence avatar use, and the highest levels among teachers without prior experience. Key facilitators included technical resources, organizational support, leadership endorsement, and training. The interviews with parents illustrated the potential role of telepresence avatars in maintaining academic and social participation during treatment, while also pointing to practical challenges such as technical instability, unclear responsibilities, and administrative procedures.

**Conclusion:**

Telepresence avatars have the potential to support the social and academic participation of children with cancer during school absences. However, successful implementation seems to depend on contextual and organizational conditions rather than on the technology itself.

**Clinical Trials Register:**

drks.de, Identifier: DRKS00036745 (registered on May 15, 2025).

## 1 Introduction

Each year, approximately 400,000 children and adolescents worldwide are diagnosed with cancer (1). A cancer diagnosis during childhood or adolescence can profoundly affect quality of life, including physical and mental health, social relationships, and academic functioning (2, 3). Prolonged school absences caused by hospitalization, immunosuppression, and treatment-related side effects frequently result in academic disruption, reduced peer contact, and social isolation (2). Survivors are also at increased risk of repeating a school year (4). Reduced school participation and school belonging are associated with poorer mental health, lower self-esteem, reduced quality of life, and difficulties in illness adjustment (2). From a developmental perspective, childhood cancer can be conceptualized as a potentially traumatic medical stressor, as prolonged stress and the disruptions of everyday life can threaten emotional, social, and educational development, with outcomes shaped by contextual and protective factors (5).

Educational participation and school belonging are key protective factors against the negative effects of prolonged school absence (6). Higher school belonging is associated with lower psychological distress, better academic outcomes, and more successful reintegration following treatment (7–9). However, children with chronic illnesses often report reduced school belonging and more negative attitudes toward school, which may further impede academic achievement and social development (9). The impact of cancer-related school absence extends to families, who not only provide medical and emotional care, but also coordinate with schools to ensure educational continuity. Families therefore face the dual burden of managing treatment-related demands while striving to maintain educational participation and social inclusion despite prolonged school interruptions (10, 11).

Telepresence systems, such as avatars, represent a promising approach to maintaining school participation during treatment-related absence (6, 12, 13). These systems act as audiovisual proxies, allowing children to attend classes virtually and interact with teachers and peers despite physical absence—an experience conceptualized as “being apart together” (6). Initial international studies report positive effects on social inclusion, emotional wellbeing, and academic engagement (6, 14, 15). A scoping review, primarily including studies from the United States, found that telepresence technologies supported connectedness, autonomy, and academic participation, thereby reducing loneliness and facilitating reintegration following school absence (13). Simultaneously, implementation challenges were identified, including technical difficulties, privacy concerns, and varying levels of acceptance among teachers and peers.

International qualitative findings further underscore the context-dependent nature of telepresence avatar use in schools. Across studies, successful implementation is linked to the interaction of child-related factors, school conditions, technical infrastructure, and stakeholder support. While telepresence avatars can enhance school connectedness and learning continuity, effective integration often increases demands on teaching staff and requires adaptations of classroom routines (6, 16).

Despite promising findings, empirical evidence remains limited. Research is largely small-scale and exploratory, focusing mainly on the perspectives of students and teachers, with parents less frequently included. As noted by Neumann et al., large-scale studies integrating multiple stakeholder perspectives and examining individual experiences and broader implementation patterns are lacking (13). More in-depth research is therefore needed to identify barriers and facilitators of telepresence avatar use in schools and to inform scalable, evidence-based implementation strategies supporting educational continuity and social participation (6, 13, 17).

## 2 Methods

### 2.1 Study design

A cross-sectional teacher survey with an exploratory qualitative parent-interview component was employed to provide a more comprehensive understanding of the barriers and facilitators of telepresence avatar use from the perspectives of teachers and affected families. In the survey component, teachers completed a questionnaire designed to assess perceived barriers and facilitators to telepresence avatar use in school. In the exploratory interview component, interviews with parents whose children had used the avatar were conducted.

### 2.2 Survey recruitment and response metrics

The study was conducted as a cross-sectional, web-based survey. Results are reported in accordance with relevant items of the Checklist for Reporting Results of Internet E-Surveys (CHERRIES) (18). The target population comprised teachers working at German schools. The survey was administered online using LimeSurvey. Recruitment was conducted at the school level and recruitment e-mails were sent between May 15 and May 22, 2025, to the official e-mail addresses of 5,019 schools in the German federal states of Schleswig-Holstein, Lower Saxony, Berlin, and Saxony. Although recruitment e-mails were sent only to schools in those four federal states, the survey link was open and could be forwarded by schools or individual teachers; however, the exact forwarding pathways could not be tracked. The survey remained open for four weeks, and no reminder e-mails were sent. School e-mail addresses were obtained from a publicly available dataset of all schools in Germany. Schools were asked to forward the invitation to all teachers in their respective school. Because the invitation was distributed via schools and internal forwarding procedures were not controlled by the research team, the exact number of individual teachers who received the invitation is unknown. Accordingly, an individual-level response rate could not be calculated. The survey was open and access to the questionnaire was provided via a survey link and QR code. Participation was voluntary and no incentive was offered. Before starting the questionnaire, informed consent was obtained electronically. The study was approved by the ethical committee of the Otto-von-Guericke University Magdeburg (184-24).

A total of 383 participants started the questionnaire, and 298 completed it (final analytical sample), corresponding to a completion rate of 77.8% among those who started the survey. Due to the anonymous survey design, IP addresses and cookies to prevent multiple participation were not used. Participants with missing demographic information were retained in the analysis and reported as “missing” where applicable. For item-level analyses, pairwise deletion was applied.

For the interviews, 10 parents of children with cancer whose children had used a telepresence avatar at school during their illness and treatment were recruited through children's cancer support associations in Northern Germany. Only *N* = 3 families met the inclusion criteria (a child diagnosed with cancer, completion of at least six months of telepresence avatar use in the school context, and no documented disease recurrence at the time of participation).

### 2.3 Measures

#### 2.3.1 Questionnaire

The questionnaire was purpose-developed for the present study because, to our knowledge, no validated German-language instrument was available to assess perceived school-based implementation barriers and facilitators of telepresence avatar use for children with cancer. Item development was informed by prior research on telepresence avatar use in schools, school participation of children with chronic illness, and implementation challenges related to digital technologies in educational and health-related contexts.

The questionnaire comprised a total of 42 items, including seven questions on demographic characteristics (gender, age, professional experience, school type, experience with pediatric cancer, experience with telepresence avatars, and digital technology affinity). The barrier items were assessed through 25 statements rated on a five-point Likert scale (“completely agree” to “completely disagree”). The full verbatim questionnaire is provided in Supplementary Material S1. The items were conceptually informed by the Consolidated Framework for Implementation Research [CFIR; (19)] and the Non-adoption, Abandonment, Scale-up, Spread, and Sustainability Framework [NASSS; (20)]. CFIR was used to guide the identification of implementation determinants related to the innovation, inner and outer setting, individuals, and implementation process. NASSS was used to capture the complexity of adopting and sustaining a technology-supported intervention situated across school and family contexts. Accordingly, the items were organized into six conceptually derived domains: technical, organizational, didactic, social, data protection, and personal barriers. The six domains were used to structure item development and descriptive reporting. They were not intended to represent a fully validated latent factor structure. Because the questionnaire was developed for descriptive implementation-oriented purposes and the six domains were conceptually derived rather than intended as empirically validated latent subscales, we did not interpret the domain structure as a validated factor structure. A conceptual mapping of all items to CFIR constructs and NASSS domains is provided in Supplementary Table S2. Internal consistency of the 25-item overall barrier index was high (Cronbach's *α* = 0.93). However, given the heterogeneous and conceptually derived nature of the items, this coefficient should be interpreted as evidence of sufficient coherence for descriptive aggregation, rather than as evidence of a validated unidimensional scale. Domain-level reliability coefficients are reported in Supplementary Table S3 as preliminary descriptive indicators.

Additionally, 10 ranking questions addressed potential facilitators. Items assessing facilitators for successful telepresence avatar use were presented as ranking tasks within each domain (technical, organizational, and external facilitators). As rankings represent forced-choice ordinal data, internal consistency was not computed.

### 2.4 Data analyses

Descriptive statistics were estimated for demographic variables as well as perceptions of barriers and facilitators of telepresence avatar use across the full sample. As barrier items were assessed using 5-point Likert scales and group sizes were unequal, non-parametric group comparisons were conducted. In addition to item-level analyses, a composite barrier score was calculated across all 25 barrier items. The composite score was used as a pragmatic overall index of perceived implementation barriers across domains, allowing group-level comparisons of general barrier perception while avoiding interpretation of the conceptually derived domains as validated latent factors. Differences between four experience groups (no prior experience; experience only with pediatric cancer; experience only with telepresence avatars; experience with both pediatric cancer and telepresence avatars) were analyzed using Kruskal–Wallis tests. When the omnibus test was statistically significant, Dunn–Bonferroni-adjusted *post hoc* pairwise comparisons were performed. Effect sizes were calculated using Eta-squared (*η*^2^) for omnibus tests and *r* for pairwise comparisons. Because the subgroup of teachers with experience only with telepresence avatars was small (*n* = 13), analyses involving this subgroup were considered exploratory. To assess the robustness of the experience-related findings, a sensitivity analysis was conducted in which this subgroup was excluded. The Kruskal–Wallis test and Dunn–Bonferroni-adjusted *post hoc* comparisons were then repeated for the remaining three experience groups: no prior experience, experience only with pediatric cancer, and combined experience with pediatric cancer and telepresence avatars (Supplementary Analysis A1). All statistical analyses were conducted using SPSS versions 29.0.0.0 and 29.0.2.0. A two-sided significance level of *p* < 0.05 was applied.

### 2.5 Interview component

Given the small number of interviews, the qualitative component was treated as exploratory and illustrative rather than as a stand-alone qualitative study designed to achieve thematic saturation. Guided problem-centered interviews were conducted to explore parents' experiences with the use of a telepresence avatar in the school context during their child's cancer treatment. The interviews were carried out online via the data protection–compliant video conferencing platform WebEx with three parents whose children had used the avatar during treatment and lasted approximately 20–30 min each. Interviews were audio-recorded using a non-internet-enabled device, transcribed according to established transcription rules (21), and pseudonymized to ensure confidentiality. Data were analyzed using qualitative content analysis following Mayring (22). Categories were developed inductively from the material based on predefined selection criteria and levels of abstraction. Initial coding was conducted by one researcher and subsequently reviewed by a second researcher. Discrepancies or ambiguities were discussed until consensus was reached. Data management and analysis were supported using MAXQDA software.

## 3 Results

### 3.1 Sample characteristics

The survey sample comprised *N* = 298 teachers (68.5% female, 27.2% male, 4.4% other or missing). Participants varied widely in terms of age, teaching experience, school type and federal state. Detailed participant characteristics are presented in Table 1.

**Table 1:** Participants’ characteristics in the teacher-survey sample (*N* = 298).

Characteristic	*n* (%)
Age group	
<30 years	8 (2.7)
30–39 years	61 (20.5)
40–49 years	90 (30.2)
50–59 years	104 (34.9)
≥ 60 years	33 (11.1)
Gender	
Female	204 (68.5)
Male	81 (27.2)
Other	3 (1.0)
Missing	10 (3.4)
Federal state	
Schleswig-Holstein	136 (46.1)
Lower Saxony	102 (34.6)
Saxony	43 (14.6)
Berlin	8 (2.7)
Saxony-Anhalt	3 (1.0)
Hamburg	2 (0.7)
Brandenburg	1 (0.3)
Years of teaching experience	
<5 years	20 (6.8)
5–15 years	87 (29.4)
16–25 years	93 (31.4)
>25 years	96 (32.4)
School type	
Elementary School	65 (21.8)
Special Education Center	36 (12.1)
Comprehensive School	68 (22.8)
High School	82 (27.5)
Vocational school	37 (12.4)
Other type of school or missing	44 (14.8)
Experience with pediatric cancer and/or telepresence avatars	
Experience only with pediatric cancer	74 (24.8)
Experience only with telepresence avatars	13 (4.4)
Experience with pediatric cancer and telepresence avatars	57 (19.1)
No experience with either	153 (51.3)

Percentages may not sum to exactly 100% due to missing data. Multiple selections were possible for the type of school.

### 3.2 Perceived barriers to telepresence avatar use

Descriptive statistics for all 25 barrier items across the total sample and stratified by experience profile are presented in Table 2. In the full sample, mean barrier ratings ranged from 2.25 (class acceptance) to 3.60 (responsibility for the telepresence avatar), with an overall mean of 3.02 on a 5-point Likert scale, indicating moderate levels of perceived implementation barriers. The highest-rated barriers concerned responsibility for the telepresence avatar (*M* = 3.60, *SD* = 1.32), internet connectivity (*M* = 3.51, *SD* = 1.37), performance assessment (*M* = 3.50, *SD* = 1.22), short-term lesson planning adjustments (*M* = 3.46, *SD* = 1.33), and data protection (*M* = 3.37, *SD* = 1.35).

**Table 2:** Descriptive statistics for perceived barriers to the use of telepresence avatars in schools, reported for the total sample (*N* = 298), and stratified by no prior experience with pediatric cancer or telepresence avatar use (*n* = 153), only pediatric cancer (*n* = 74), only telepresence avatar use (*n* = 13) and experience with pediatric cancer and telepresence avatar use (*n* = 57).

Barrier	Total sample *M* (*SD*)	No experience *M (SD)*	Experience only with cancer *M* (*SD*)	Experience only with telepresence avatars *M* (*SD*)	Experience with cancer and telepresence avatars *M* (*SD*)
Technical barriers					
Internet connectivity	3.51 (1.37)	3.58 (1.32)	3.39 (1.42)	3.10 (1.50)	3.53 (1.40)
Device compatibility	3.31 (1.33)	3.57 (1.66)	3.33 (1.33)	2.60 (1.60)	2.43 (1.26)
Technical susceptibility	3.28 (1.16)	3.47 (1.23)	3.27 (1.15)	3.10 (1.34)	2.71 (1.14)
Own technical competence	2.79 (1.25)	3.03 (1.23)	2.81 (1.28)	2.20 (1.21)	2.12 (1.04)
Organizational barriers					
Responsibility for telepresence avatar	3.60 (1.32)	3.92 (1.17)	3.61 (1.31)	2.90 (1.36)	2.90 (1.35)
Short-term changes	3.46 (1.33)	3.77 (1.20)	3.09 (1.48)	3.00 (1.55)	3.04 (1.21)
Provision of digital teaching material	3.31 (1.29)	3.45 (1.20)	3.03 (1.43)	3.10 (1.38)	3.00 (1.25)
Communication with parents	3.09 (1.25)	3.18 (1.21)	2.81 (1.20)	2.70 (1.59)	2.71 (1.28)
Transport of telepresence avatar between rooms	3.05 (1.29)	3.25 (1.86)	2.95 (1.31)	2.30 (1.38)	2.67 (1.41)
Collegial support	2.91 (1.29)	3.18 (1.26)	2.73 (1.28)	3.10 (1.59)	2.33 (1.18)
Preparation time	2.60 (1.16)	2.78 (1.17)	2.34 (1.05)	2.00 (1.28)	2.20 (1.14)
Didactic barriers					
Performance assessment	3.50 (1.22)	3.66 (1.21)	3.28 (1.23)	3.80 (1.32)	3.29 (1.18)
Interaction with child with cancer	2.92 (1.15)	2.99 (1.11)	2.78 (0.15)	2.70 (1.54)	2.71 (1.02)
Communication through telepresence avatar	2.92 (1.10)	3.05 (1.08)	2.81 (1.11)	2.70 (1.11)	2.49 (1.01)
Integration in classroom	2.88 (1.16)	3.08 (1.14)	2.66 (1.16)	2.40 (1.19)	2.39 (1.00)
Distraction of class	2.88 (1.11)	3.19 (1.11)	2.69 (1.02)	2.20 (1.12)	2.18 (0.83)
Social barriers					
Emotional stress (child with cancer)	2.94 (1.13)	3.09 (1.11)	2.86 (1.03)	3.20 (1.18)	2.59 (1.22)
Distance to teacher	2.87 (1.11)	2.86 (1.16)	2.95 (1.09)	3.10 (1.07)	2.55 (0.93)
Emotional stress (class)	2.37 (1.00)	2.53 (1.03)	2.31 (0.96)	2.20 (0.83)	2.04 (0.89)
Class acceptance	2.25 (1.10)	2.45 (1.17)	2.00 (0.97)	2.50 (0.97)	1.88 (0.90)
Data protection barriers					
Data protection	3.37 (1.35)	3.46 (1.28)	3.33 (1.40)	2.60 (1.33)	2.94 (1.31)
Parent approval	3.22 (1.28)	3.37 (1.19)	3.31 (1.35)	2.60 (1.26)	2.71 (1.32)
Teacher approval	2.98 (1.32)	3.18 (1.22)	3.03 (1.39)	2.80 (1.13)	2.35 (1.30)
Personal barriers					
Workload	2.98 (1.21)	3.26 (1.17)	2.61 (1.17)	2.50 (1.05)	2.53 (1.19)
Openness to technology	2.55 (1.26)	2.82 (1.25)	2.38 (1.30)	2.30 (1.04)	2.12 (1.19)
Overall mean					
Overall mean	3.02 (0.73)	3.23 (0.64)	2.93 (0.78)	2.72 (0.71)	2.62 (0.67)

Barriers were rated on a 5-point Likert scale from 1 (“strongly disagree”) to 5 (“strongly agree”). Higher values indicate stronger perceived barriers.

### 3.3 Group differences

Kruskal–Wallis analyses revealed significant differences between the four experience groups for most perceived barrier domains (Table 3). Significant differences between experience profiles were observed for the composite score [*H*(3) = 28.08, *p* < .001, *η*^2^ = 0.08]. Teachers without prior experience reported the highest overall barrier levels (*M* = 3.23), whereas teachers with combined experience in pediatric cancer and telepresence avatar use reported the lowest levels (*M* = 2.62). *Post hoc* comparisons showed significantly lower overall barrier scores among teachers with combined experience compared to those without experience (*p* < .001, *r* = 0.35). Because the subgroup with experience only with telepresence avatars was small (*n* = 13), a sensitivity analysis excluding this group was conducted. The overall pattern remained consistent (Supplementary Analysis A1). At the item level, Kruskal–Wallis analyses revealed significant group differences for perceived technical competence, technical susceptibility, device compatibility, preparation time, responsibility for the avatar, short-term lesson adaptations, communication with parents, transport of the avatar between rooms, collegial support, classroom integration, communication via the avatar, distraction of the class, emotional stress of the class, class acceptance, data protection concerns, approval by teachers and parents, perceived workload, and openness to technology, with effect sizes generally small to moderate. The largest group difference was found for perceived class distraction (*η*^2^ = 0.12). No significant group differences emerged for internet connectivity, provision of digital teaching material, performance assessment, interaction with the child with cancer, emotional stress of the child with cancer, or perceived distance to the teacher. Overall, the pattern indicates a graded association between reported experiential familiarity and lower perceived implementation barriers within this self-selected survey sample.

**Table 3:** Kruskal–Wallis analyses for group differences in perceived barriers to telepresence avatar use (item-level and overall composite score) across four experience profiles, using participants with non-missing experience-group data (*N* = 297).

Barrier	*H*	*df*	*p*	*η*^2^ (*f*)	no experience vs. experience only with cancer [adj. *p* (*r*)]	no experience vs. experience only with telepresence avatars [adj. *p* (*r*)]	no experience vs. experience with cancer and telepresence avatars [adj. *p* (*r*)]
Technical barriers							
Internet connectivity	1.645	3	.649	n.a.	n.a.	n.a.	n.a.
Device compatibility	32.857	3	<.001*	0.10 (0.33)	1.000	.179	<.001* (0.38)
Technical susceptibility	20.606	3	<.001*	0.06 (0.25)	1.000	.317	<.001* (0.32)
Own technical competence	20.179	3	<.001*	0.06 (0.25)	1.000	.102	<.001* (0.24)
Organizational barriers							
Responsibility for telepresence avatar	29.472	3	<.001*	0.09 (0.31)	.485	.019* (0.23)	<.001* (0.34)
Short-term changes	18.399	3	<.001*	0.05 (0.24)	.018* (0.20)	.556	<.001* (0.26)
Provision of digital teaching material	5.986	3	.112	n.a.	n.a.	n.a.	n.a.
Communication with parents	8.456	3	.037*	0.02 (0.14)	.157	.987	.149
Transport of telepresence avatar between rooms	11.473	3	.009*	0.03 (0.17)	1.000	.066	.060
Collegial support	13.766	3	.003*	0.04 (0.20)	.482	1.000	.003* (0.25)
Preparation time	11.636	3	.009*	0.03 (0.18)	.213	.226	.038* (0.19)
Didactic barriers							
Performance assessment	4.666	3	.198	n.a.	n.a.	n.a.	n.a.
Interaction with child with cancer	3.581	3	.310	n.a.	n.a.	n.a.	n.a.
Communication through telepresence avatar	12.007	3	.007*	0.03 (0.18)	.368	1.000	.005* (0.23)
Integration in classroom	16.986	3	<.001*	0.05 (0.23)	.063	.703	.001* (0.26)
Distraction of class	39.236	3	<.001*	0.12 (0.37)	.003* (0.20)	.048* (0.15)	<.001* (0.33)
Social barriers							
Emotional stress (child with cancer)	7.523	3	.057	n.a.	n.a.	n.a.	n.a.
Distance to teacher	5.311	3	.150	n.a.	n.a.	n.a.	n.a.
Emotional stress (class)	9.796	3	.020*	0.02 (0.15)	1.000	1.000	.014* (0.21)
Class acceptance	16.681	3	<.001*	0.05 (0.23)	.072	1.000	.003* (0.25)
Data protection barriers							
Data protection	12.724	3	.005*	0.03 (0.19)	.949	.030* (0.22)	.064
Teacher approval	18.373	3	<.001*	0.05 (0.24)	1.000	.737	<.001* (0.29)
Parent approval	16.527	3	<.001*	0.05 (0.23)	1.000	.132	.003* (0.25)
Personal barriers							
Workload	22.065	3	<.001*	0.07 (0.27)	.002* (0.24)	.100	.003* (0.24)
Openness to technology	11.439	3	.010*	0.03 (0.18)	.070	.358	.075
Overall mean							
Overall mean	28.079	3	<.001*	0.09 (0.31)	.048* (0.18)	.261	<.001* (0.35)

Adj. *p* = Dunn–Bonferroni-adjusted *p*; H = Kruskal–Wallis test statistic; *η*^2^ = eta-squared effect size; Cohen's *f* was derived from *η*^2^ using *f* = √[*η*^2^/(1 − *η*^2^)]; *r* = pairwise effect size for Dunn–Bonferroni *post hoc* comparisons, calculated as *r* = |z|/√*N*. Pairwise *r* values are reported in parentheses for statistically significant *post hoc* comparisons. *η*^2^, Cohen's *f*, and *post hoc* comparisons are reported only for items with a statistically significant omnibus Kruskal–Wallis test.

* *p* < 0.05.

### 3.4 Perceived facilitators to telepresence avatar use

Table 4 presents the rankings of facilitators across three domains: technical, organizational, and external factors. Teachers most frequently identified technical facilitators as the most important conditions for successful implementation. Specifically, good technical equipment was selected as the top facilitator by 57.7% of participants, followed by technical support (22.5%) and sufficient technical expertise among staff (14.8%). Within the organizational domain, teachers showed a more distributed pattern of preferences. The factors most frequently ranked as most important included effective school organization (20.1%), support from school leadership (15.1%) and good communication with parents (14.8%). Regarding external facilitators, formal introduction and training on avatar use was ranked as the top facilitator by 69.8% of respondents.

**Table 4:** Descriptive statistics for perceived facilitators to the use of telepresence avatars in schools, reported for the total sample (*N* = 298).

Technical facilitators	Rank 1 (%)	Rank 2 (%)	Rank 3 (%)
Good technical equipment	57.7	22.5	14.8
Technical support	19.8	42.3	32.9
Good technical expertise	17.4	30.2	47.3
Organizational facilitators	Rank 1	Rank 2	Rank 3
School organization	20.1	17.1	15.8
Support from school management	15.1	11.4	11.1
Good communication with parents	14.8	20.1	19.8
Agreements among teaching staff	12.8	21.1	18.1
Compensation (e.g., reduction in hours)	10.4	3.7	6.4
Best practice examples	9.7	9.4	8.1
Support with lesson planning using telepresence avatar	8.1	10.4	10.7
Integration into teacher training	6.0	3.4	6.7
External facilitators	Rank 1	Rank 2	Rank 3
Introduction to the use of telepresence avatars in schools (e.g., training)	69.8	28.2	n.a.
Permanent contact persons in the company	28.2	68.5	n.a.

Facilitators were ranked by the participants according to their importance. Percentages may not sum to exactly 100% due to missing data or the item not being chosen within the first three ranks.

n.a., not applicable.

### 3.5 Parent interview findings

The interview sample comprised three parents of children with cancer whose children had used a telepresence avatar in a school context during treatment. Due to the exploratory nature of the pilot study, only limited sociodemographic information was collected for the qualitative sample. These parents were interviewed using qualitative interviews. The interviews provide illustrative insights into how avatar implementation was experienced by three families and are used to contextualize the teacher-survey findings.

All parents reported that their child's cancer diagnosis resulted in a complete or prolonged interruption of regular school attendance. In this context, the avatar was perceived as a means to reduce isolation and maintain a minimum level of school and social participation. One parent stated: “*She wasn’t in school at all. Yes, during therapy. That was six months for us.”* (A4S_P_03).

Access to the avatar was not initiated by the parents themselves but facilitated by external institutions, including hospital social services, support associations, and organizations such as the German Cancer Society. This external support was described as essential for both introducing the avatar and relieving families. One parent noted: “*The German Cancer Society, which lends out the avatars […] they did everything.”* (A4S_P_03).

Further organization and coordination of avatar use depended strongly on the commitment of individual actors. Parents particularly emphasized the role of clinic staff and supportive teachers in facilitating implementation. Where responsibilities were clearly defined, the process was described as smooth: “*One employee […] organized everything, and the next day we started using the avatar.”* (A4S_P_03).

Regarding usage, two parents reported that the avatar was used almost daily, although frequency depended on the child's daily condition and treatment demands. This allowed for continuous, albeit sometimes unstable, contact with the school.

Across the three interviews, parents described positive experiences of telepresence avatar use. The avatar enabled ongoing participation in class despite physical absence and supported social interaction with classmates, which was described as particularly important during breaks or informal moments: “*a little company”* (A4S_P_03). Parents also emphasized the prevention of learning deficits: “*No learning deficit […] that was important.”* (A4S_P_02). Additionally, fixed lesson times were reported to support daily structure during illness.

At the same time, substantial barriers were identified. All parents described technical problems, particularly unstable internet connections, as stressful: “*The biggest problem was the Wi-Fi coverage at school.”* (A4S_P_01). Two parents reported significant delays due to lengthy data protection and consent procedures: “*All students needed a consent form […] that took a long time.”* (A4S_P_03). In one case, individual teachers explicitly refused to allow the telepresence avatar in their lessons.

Parents suggested several improvements, including better technical equipment and simplified administrative processes. They specifically mentioned enhanced technical integration (e.g., direct connection to digital whiteboards) and a reduction in the burden of data protection procedures.

In these three cases, telepresence avatars were described as valuable tools for maintaining social and academic participation during cancer treatment. Despite recurring technical and organizational challenges, positive experiences outweighed negative ones. However, stable technical infrastructure, clear organizational responsibilities, and transparent data protection regulations were identified as critical prerequisites for sustainable implementation.

## 4 Discussion

This study investigates teachers' perceptions of barriers and facilitators to implementing telepresence avatars for children with cancer during treatment-related school absences, complemented by parent interviews to contextualize implementation experiences. Overall, the findings corroborate and extend prior international research by indicating that successful implementation depends less on the technology itself than on contextual conditions (13).

### 4.1 Interpretation of the findings

Social participation and school integration are key factors for the well-being of children with cancer, but are limited by practical barriers (13). Overall, teachers recognized the potential of telepresence avatars as tools enabling continued school participation and classroom connection during longer absences. At the same time, they reported several practical barriers that may hinder implementation in everyday school practice. Technical and organizational barriers were identified as the most relevant challenges in school practice. Concerns primarily related to responsibility for the telepresence avatar, technical reliability, data protection, and flexibility within classroom routines. These findings are consistent with international research showing that uncertainties around accountability, infrastructure, and institutional routines frequently hinder implementation (13, 16, 23).

The exploratory parent interviews provide illustrative context for why successful implementation may matter from the family perspective. Parents perceived telepresence avatars as particularly valuable in mitigating social isolation during treatment-related absences. The opportunity to participate in lessons and class activities was described as fostering feelings of normality and class affiliation, which parents regarded as important for their children's everyday well-being. These accounts illustrate the potential psychosocial relevance of virtual school attendance and align with previous research highlighting the role of telepresent school participation as a stabilizing and normalizing factor for children with chronic illnesses (24).

A central survey finding concerns the association between reported experiential familiarity and perceived barriers. Teachers with combined experience in pediatric cancer and telepresence avatar use reported the lowest overall barrier levels, whereas teachers without prior experience reported the highest levels. The sensitivity analysis excluding the small subgroup of teachers with experience only with telepresence avatars supported the overall pattern. However, this analysis addresses only the influence of subgroup composition and does not account for potential selection bias in survey participation or in who had prior experience in the first place. The comparatively high proportion of respondents reporting experience with pediatric cancer and/or telepresence avatars, given the rarity of pediatric cancer in routine teaching careers, suggests possible self-selection toward teachers with a personal or professional stake in the topic. Accordingly, the association between prior experience and lower perceived barriers should be interpreted cautiously and not causally. Because of the cross-sectional design, the direction of the association cannot be determined. Experience may have contributed to a more realistic and less anticipatory appraisal of implementation demands. Conversely, teachers with lower baseline concerns, greater openness to technology, stronger institutional support, better digital infrastructure, or links to external support organizations may have been more likely to become involved in avatar use in the first place. Nevertheless, the observed differences may reflect varying levels of implementation readiness, with hands-on experience potentially helping to reduce uncertainty and normalize avatar use within school routines. This interpretation is consistent with Neumann et al. (13) and with Johannesen et al.'s (23) concept of “multi-site domestication,” whereby technologies gradually become embedded within established practices.

Regarding facilitators, teachers emphasized reliable equipment, accessible technical support, clear internal structures, leadership endorsement, and formal training. This reflects findings from Fletcher & Bond (25) and Fletcher et al. (26) who identified training and practical support as enabling conditions. Pedagogical integration was considered relevant but secondary. This suggests that telepresence avatars are perceived primarily as organizational and technical innovations rather than pedagogical tools. Overall, the findings suggest that teachers' perceived implementation challenges may be experience-sensitive and structurally shaped, rather than inherent to the avatar technology itself. From an implementation perspective, the findings underscore that telepresence avatars should not be introduced as *ad hoc* solutions but as part of more refined implementation frameworks, as also suggested by Neumann et al. (13).

The exploratory parent interviews further support this interpretation. Parents' accounts suggested that the benefits of telepresence avatar use depended strongly on stable technical conditions, clear communication with the school, and the willingness of teachers and classmates to integrate the avatar into everyday classroom life. Thus, the parent interviews help illustrate the practical consequences of the barriers identified in the teacher survey: when technical and organizational conditions are not sufficiently addressed, the potential of telepresence avatars to support participation may remain only partially realized.

Taken together, the teacher survey and the illustrative parent accounts suggest that virtual school attendance may hold substantial potential to support children with cancer, while structural and technical barriers hinder its seamless integration into everyday school practice. In particular, unreliable Wi-Fi connections were identified as a key barrier to the implementation of telepresence avatars from both perspectives.

### 4.2 Classification in the research context

This study contributes to the growing literature on telepresence avatars as instruments for maintaining educational participation of children with chronic illnesses. Prior research shows that prolonged cancer-related school absences are associated with academic and psychosocial risks, highlighting the importance of school reintegration as part of psychosocial care (10, 27, 28). Meta-analytic evidence indicates that school reintegration programs improve academic and psychosocial outcomes (29). Recent research suggests that telepresence avatars can support continued classroom participation and social connectedness during extended absences, while also introducing additional demands on families (6, 13, 15, 17). The present findings extend this literature by systematically identifying concrete school-related conditions that may facilitate or hinder implementation. In line with previous research, technical reliability, data protection regulations, and organizational clarity emerged as central prerequisites for successful implementation (16, 30, 31). Teachers frequently reported uncertainty regarding consent procedures, accountability in case of technical failure, and institutional support. Data protection emerged as a central barrier across both data sources, constituting a significant obstacle in the school setting. This parallels challenges well documented in telemedicine, where organizational and bureaucratic constraints repeatedly hinder the translation of innovative care models into routine practice (32), and mirror results from Germany and Japan showing that unclear legal frameworks and a lack of standardized procedures can significantly delay or prevent implementation, with statements such as “*nothing like this has ever been done before*” (30). Importantly, the observed association between prior experience and lower perceived barriers is consistent with research emphasizing the relevance of training, ongoing support, and implementation readiness, although the cross-sectional design does not allow causal conclusions about the effect of experience itself (25, 26). Schools that provide structured guidance, technical assistance, and leadership endorsement are possibly better equipped to integrate telepresence avatars into daily routines. From a broader perspective, this study can also be situated within the research on inclusive education and the right to educational participation for children with chronic illnesses. Telepresence avatars represent one possible means of enabling participation in school life despite physical absence, thereby supporting continuity of education and social interaction (33).

### 4.3 Limitations

Recruitment was conducted at the school-level and internal forwarding to individual teachers could not be tracked. Therefore, an individual-level response rate could not be calculated, and respondent and non-respondent characteristics could not be compared. Participation was voluntary, which may have introduced substantial self-selection bias. In particular, the comparatively high proportion of teachers reporting experience with pediatric cancer and/or telepresence avatars suggests that experienced teachers may have been more likely to participate which limits representativeness. In addition, not all German federal states were represented, restricting generalizability. The cross-sectional design precludes causal interpretation, and school-level confounding and survivorship bias are also possible. Group sizes differed across experience profiles, and the subgroup with experience only with telepresence avatars was small. Findings involving this subgroup should therefore be interpreted as exploratory, although sensitivity analyses supported the overall pattern. The parent interviews were based on a very small exploratory sample of three families and should therefore be interpreted only as illustrative contextual material. Finally, although internal consistency of the overall barrier index was high, the questionnaire was purpose-developed and has not yet undergone comprehensive psychometric validation; therefore, the composite score should be interpreted as a descriptive implementation-oriented index rather than as a validated latent construct, and future studies should further examine the instrument's validity and reliability.

### 4.4 Implications for future research and practice

Several implications can be derived from the findings. First, schools and policymakers should approach telepresence avatar use as an institutional responsibility rather than an individual solution managed by single teachers. Clear procedures, defined responsibilities, reliable technical support, and transparent consent processes are essential to reduce uncertainty and workload. In addition, bureaucratic hurdles, particularly in data protection, should be streamlined. The association between experiential familiarity and lower perceived barriers suggests that structured training and guided hands-on experiences may be promising strategies to address anticipatory concerns. One potential strategy is the systematic integration of digital participation tools for students with chronic illnesses into pre-service teacher education and professional development programs in order to facilitate sustainable implementation and improve educational continuity for affected children. At the system level, stronger coordination between schools, educational authorities, and health-care stakeholders is required to establish sustainable responsibility and financing models. Without such structures, telepresence avatar use risks remaining dependent on individual commitment and external support.

Future research should extend these implementation-focused findings by systematically examining how digitally mediated school participation can be integrated into existing systems. Longitudinal and fully integrated mixed-methods designs would be particularly valuable for examining how telepresence avatar implementation develops over time, how perceived barriers change with experience, how effective targeted training is and which support structures are most effective in sustaining use across different school contexts. Furthermore, future research should explore how digital participation affects not only chronically ill students themselves but also the systems in which they are integrated (e.g., dynamics of the class community, teachers). Finally, future research should also consider technical developments, for example the extent to which newer robotic systems with enhanced interaction capabilities can further improve participation without compromising data protection or simplicity.

In summary, this study demonstrates that the successful school-based use of telepresence avatars for children with cancer is shaped by contextual factors, including organizational readiness, technical reliability, data protection aspects, and teachers' reported experiential familiarity, although the latter should be interpreted cautiously in light of possible self-selection. By identifying teacher-reported barriers and facilitating conditions and supplementing them with illustrative parent accounts, the study provides an important foundation for structured, sustainable implementation within inclusive educational practice and offers a promising starting point for future longitudinal research on effective implementation strategies.

## Data Availability

The raw data supporting the conclusions of this article will be made available by the authors, without undue reservation.
