# Supplementary material for: Telepresence avatar use for children with cancer in schools: a study on barriers and facilitators
Source: Front Digit Health. 2026 Jul 9;8:1867390. doi: 10.3389/fdgth.2026.1867390 (PMC13391852; doi:10.3389/fdgth.2026.1867390)
Supplement: Supplementary file 1 [file Datasheet1.docx]

**Supplementary Table S1.** Full questionnaire for teachers on barriers and facilitators of school-based use of telepresence avatars

| **Technical Barriers** Which of the following technical aspects could, in your opinion, represent challenges in the school-based use of avatars?  Instruction: Please select the applicable answer for each item. |  |
| --- | --- |
| **Item** | **Response options** |
| Internet connection | - Do not agree at all - Rather do not agree - Party agree - Rather agree - Fully agree |
| Own technical competence | - Do not agree at all - Rather do not agree - Party agree - Rather agree - Fully agree |
| Technical susceptibility to malfunction of the avatar | - Do not agree at all - Rather do not agree - Party agree - Rather agree - Fully agree |
| Device compatibility (e.g., with other technical devices at school) | - Do not agree at all - Rather do not agree - Party agree - Rather agree - Fully agree |
| **Organizational Barriers**  Which of the following organizational aspects could, in your opinion, represent challenges in the school-based use of avatars?  Instruction: Please select the applicable answer for each item. |  |
| **Item** | **Response options** |
| Time required for lesson preparation | - Do not agree at all - Rather do not agree - Party agree - Rather agree - Fully agree |
| Time required for parent communication | - Do not agree at all - Rather do not agree - Party agree - Rather agree - Fully agree |
| Digital provision of teaching material | - Do not agree at all - Rather do not agree - Party agree - Rather agree - Fully agree |
| Taking the avatar along when changing rooms | - Do not agree at all - Rather do not agree - Party agree - Rather agree - Fully agree |
| Responsibility for the avatar | - Do not agree at all - Rather do not agree - Party agree - Rather agree - Fully agree |
| Short-term changes in everyday school life | - Do not agree at all - Rather do not agree - Party agree - Rather agree - Fully agree |
| Collegial support and acceptance | - Do not agree at all - Rather do not agree - Party agree - Rather agree - Fully agree |
| **Didactic Barriers**  Which of the following didactic aspects could, in your opinion, represent challenges in the school-based use of avatars?  Instruction: Please select the applicable answer for each item. |  |
| **Item** | **Response options** |
| Distraction of other students by the avatar | - Do not agree at all - Rather do not agree - Party agree - Rather agree - Fully agree |
| Communication via avatar | - Do not agree at all - Rather do not agree - Party agree - Rather agree - Fully agree |
| Interaction with the child with cancer | - Do not agree at all - Rather do not agree - Party agree - Rather agree - Fully agree |
| Integration of the avatar into teaching | - Do not agree at all - Rather do not agree - Party agree - Rather agree - Fully agree |
| Performance assessment | - Do not agree at all - Rather do not agree - Party agree - Rather agree - Fully agree |
| **Social Barriers**  Which of the following social aspects could, in your opinion, represent challenges in the school-based use of avatars?  Instruction: Please select the applicable answer for each item. |  |
| **Item** | **Response options** |
| Acceptance by the class community | - Do not agree at all - Rather do not agree - Party agree - Rather agree - Fully agree |
| Emotional burden on the child with cancer | - Do not agree at all - Rather do not agree - Party agree - Rather agree - Fully agree |
| Emotional burden on the class community | - Do not agree at all - Rather do not agree - Party agree - Rather agree - Fully agree |
| Distance to the teacher | - Do not agree at all - Rather do not agree - Party agree - Rather agree - Fully agree |
| **Data Protection Barriers**  Which of the following data-protection aspects could, in your opinion, represent challenges in the school-based use of avatars?  Instruction: Please select the applicable answer for each item. |  |
| Data protection regulations | - Do not agree at all - Rather do not agree - Party agree - Rather agree - Fully agree |
| Approval of teachers for use in lessons | - Do not agree at all - Rather do not agree - Party agree - Rather agree - Fully agree |
| Approval of the parents of the class community | - Do not agree at all - Rather do not agree - Party agree - Rather agree - Fully agree |
| **Personal Barriers**  Which of the following personal aspects could, in your opinion, represent challenges in the school-based use of avatars?  Instruction: Please select the applicable answer for each item. |  |
| Openness toward new technology | - Do not agree at all - Rather do not agree - Party agree - Rather agree - Fully agree |
| Workload | - Do not agree at all - Rather do not agree - Party agree - Rather agree - Fully agree |
| **Technical Facilitators**  Please rank the following technical aspects according to their importance for the successful use of avatars in the school context.  Instruction: All your answers must be different and must be assigned. Please select a maximum of 3 answers. Please number each box in order of your preference, beginning with 1 to 3. |  |
| **Rank** | **Option** |
|  | Good technical equipment |
|  | Technical support |
|  | Good own technical competence |
| **Organizational Facilitators**  Please rank the following organizational aspects according to their importance for the successful use of avatars in the school context.  Instruction: All your answers must be different and must be assigned. Please select a maximum of 8 answers. Please number each box in order of your preference, beginning with 1 to 8. |  |
| **Rank** | **Options** |
|  | Compensation (e.g., reduction of teaching hours) |
|  | School organization |
|  | Agreements within the teaching staff |
|  | Good communication with parents |
|  | Support from school leadership |
|  | Support with lesson planning using the avatar |
|  | Integration into teacher education |
|  | Best-practice examples |
| **External Support Facilitators**  Please rank the following aspects regarding external support according to their importance for the successful use of avatars in the school context.  Instruction: All your answers must be different and must be assigned. Please select a maximum of 2 answers. Please number each box in order of your preference, beginning with 1 to 2. |  |
| **Rank** | **Item** |
|  | Permanent contact persons at the company |
|  | Introduction to school-based use of the avatar (e.g., training/professional development) |

*Note***.** The questionnaire was administered in German. This table provides an English translation of the questionnaire item wording and response options from the administered survey. General introductory/consent pages are not included.

**Supplementary Table S2.** Conceptual mapping of barrier items to CFIR and NASSS frameworks

| **Barrier item** | **Questionnaire domain** | **CFIR-informed rationale** | **NASSS-informed rationale** | **Item-specific rationale** |
| --- | --- | --- | --- | --- |
| Internet connectivity | Technical | Inner setting: available resources; implementation infrastructure | Technology: material and technical features | Stable internet access is required for synchronous participation and reliable classroom connection |
| Device compatibility | Technical | Innovation: compatibility; inner setting: available resources | Technology: usability and integration with existing systems | Telepresence avatar use depends on compatibility with existing networks, and digital classroom equipment. |
| Technical susceptibility | Technical | Innovation: complexity; implementation infrastructure | Technology: reliability and technical dependability | Technical interruptions may disrupt lessons and increase perceived implementation burden. |
| Own technical competence | Technical | Individuals: knowledge and beliefs; capability | Adopters: staff knowledge and confidence | Teachers’ perceived technical competence may influence allowing the use of telepresence avatars, confidence in using and integrating the telepresence avatar in their teaching. |
| Responsibility for telepresence avatar | Organizational | Inner setting: roles, responsibilities, readiness for implementation | Organization: allocation of tasks and responsibilities | Clear responsibility is needed for setup, use, troubleshooting, and coordination. |
| Short-term changes | Organizational | Innovation: adaptability; inner setting: compatibility with workflows | Organization: disruption to routines | Change of lesson plans, room changes, or spontaneous classroom activities can pose a barrier to telepresence avatar use, since it must be taken into account additionally. |
| Provision of digital teaching material | Organizational | Implementation process: planning; available resources | Organization: work required to embed the technology | Participation through a telepresence avatar may require additional or adapted digitally provided learning materials. |
| Communication with parents | Organizational | Process: engaging; outer setting: patient/family needs | Adopters and value proposition: family-school coordination | Implementation requires coordination between teachers, parents, and external support. |
| Transport of telepresence avatar between rooms | Organizational | Inner setting: logistical infrastructure; available resources | Organization: operational workload | Moving and positioning the telepresence avatar within the school context (classrooms, schoolyard) may create practical workload and responsibility issues. |
| Collegial support | Organizational | Inner setting: implementation climate; networks and communication | Organization: collective capacity for adoption | Support from colleagues may reduce individual burden and normalize telepresence avatar use in the school setting. |
| Preparation time | Organizational | Inner setting: available resources; implementation readiness | Organization: workability and workload | Additional preparation time may affect teachers’ willingness and perceived feasibility of telepresence avatar use. |
| Performance assessment | Didactic | Innovation: compatibility with existing practices | Value proposition: perceived educational benefit and feasibility | Teachers may be uncertain how to assess performance or learning outcomes during attendance through the telepresence avatar. |
| Interaction with child with cancer | Didactic | Innovation: relative advantage and complexity; individuals involved | Adopters: interaction between child, teachers, and classmates | Interaction through telepresence avatar may differ from in-person interaction and require pedagogical adjustment. |
| Communication through telepresence avatar | Didactic | Innovation: design and complexity | Technology: communication functionality | Communication quality through the telepresence avatar may affect classroom participation and teacher-child interaction. |
| Integration in classroom | Didactic | Innovation: compatibility and adaptability | Organization: embedding technology into routine practice | The telepresence avatar must be integrated into classroom routines, seating arrangements, group work, and lesson formats. |
| Distraction of class | Didactic | Inner setting: implementation climate; compatibility with classroom routines | Adopters: responses of classmates and teachers | The avatar may affect attention, classroom management, or peer behavior. |
| Emotional stress (child with cancer) | Social | Outer setting: child/family needs; individuals involved | Condition: illness-related vulnerability and complexity | Participation may create emotional strain for the child, especially when illness and absence become apparent. |
| Distance to teacher | Social | Individuals: relationships and communication | Adopters: teacher-child relationship | Telepresence-avatar mediated participation may influence perceived teacher-student connection. |
| Emotional stress (class) | Social | Inner setting: classroom climate; individuals involved | Adopters: classmates and school community | Classmates may experience emotional strain when confronted with the child’s illness. |
| Class acceptance | Social | Inner setting: culture and implementation climate | Adopters: peer acceptance and social embedding | Peer acceptance is central for meaningful social participation and successful classroom integration. |
| Data protection | Data protection | Outer setting: policies, regulations, and external constraints | Wider system: legal, regulatory, and policy context | Telepresence avatar use may raise data privacy questions. |
| Parent approval | Data protection | Outer setting: external policy and consent requirements; engaging stakeholders | Wider system/adopters: consent and stakeholder approval | Implementation may depend on parental consent for classmates’ participation in an avatar-mediated classroom setting. |
| Teacher approval | Data protection | Inner setting: readiness and implementation climate; individuals’ beliefs | Adopters/organization: professional acceptance and institutional permission | Teachers’ willingness may influence whether they allow the telepresence avatar in their lessons. |
| Workload | Personal | Individuals: perceived capability; inner setting: available resources | Organization/adopters: work required from staff | Additional perceived workload may reduce implementation readiness and willingness to use the telepresence avatar. |
| Openness to technology | Personal | Individuals: knowledge, beliefs, and attitudes toward the innovation | Adopters: acceptance and engagement with technology | Teachers’ openness to new technology may influence perceived barriers and readiness to integrate the telepresence avatar. |

*Note*. CFIR = Consolidated Framework for Implementation Research; NASSS = Non-adoption, Abandonment, Scale-up, Spread, and Sustainability Framework. The mapping is conceptual and was used to make the item rationale transparent. It does not imply that the questionnaire constitutes a validated operationalization of CFIR or NASSS constructs.

**Supplementary Table S3.** Domain-level reliability coefficients for the barrier items.

| **Domain** | **Number of items** | **Cronbachs alpha** |
| --- | --- | --- |
| Technical barriers | 4 | α = .76 |
| Organizational barriers | 7 | α = .84 |
| Didactic barriers | 5 | α = .83 |
| Social barriers | 4 | α = .78 |
| Data protection barriers | 3 | α = .84 |
| Personal barriers | 2 | α = .62 (Inter-item correlation: *r* = .45) |

**Supplementary Analysis A1**

Because the subgroup of teachers with experience only with telepresence avatars was small (*n* = 13), a sensitivity analysis was conducted to assess the robustness of the main experience-group comparison. This subgroup was excluded, and the Kruskal–Wallis test was repeated for the remaining three groups: teachers without prior experience, teachers with experience only with pediatric cancer, and teachers with combined experience in pediatric cancer and telepresence avatar use. The three-group comparison remained statistically significant, *H*(2) = 26.73, *p* < .001, indicating that overall perceived barrier scores differed between the remaining experience groups. The effect size was η² = 0.09, suggesting a small to moderate effect. Dunn–Bonferroni-adjusted post hoc comparisons showed that teachers with combined experience in pediatric cancer and telepresence avatar use reported significantly lower overall barrier scores than teachers without prior experience (*p* < .001). Teachers with experience only in pediatric cancer also reported significantly lower overall barrier scores than teachers without prior experience (*p* = .024). The difference between teachers with experience only in pediatric cancer and those with combined experience was not statistically significant after correction (*p* = .066).
